# Pediatric sequential organ failure assessment for predicting outcomes in ECMO-bridged pediatric heart transplant recipients: experience from the largest pediatric heart transplant center in China

**DOI:** 10.3389/fmed.2025.1631616

**Published:** 2025-07-04

**Authors:** Wang-zi Li, Xian-ming Zhou, Wei Su, Cheng Zhou, Guo-hua Wang, Jia-wei Shi, Nian-guo Dong

**Affiliations:** Department of Cardiovascular Surgery, Union Hospital, Tongji Medical College, Huazhong University of Science and Technology, Wuhan, China

**Keywords:** ECMO, advanced heart failure, pediatric heart transplant, pSOFA, bridge-to-transplant

## Abstract

**Objective:**

Advanced heart failure in children sometimes requires mechanical circulatory support as a bridge to transplantation, with extracorporeal membrane oxygenation (ECMO) remaining a critical option despite its associated risks. The pediatric Sequential Organ Failure Assessment (pSOFA) may have potential in evaluating prognosis in ECMO-bridged candidates.

**Methods:**

188 Children underwent orthotopic heart transplantation in Union hospital, Tongji Medical College, Huazhong University of Science and Technology, between January 2018 and April 2025 were studied retrospectively, with 24 received ECMO assistance as a bridge to transplant. Patients were divided into two groups according to outcomes while discharged. Serial pediatric Sequential Organ Failure Assessment and other medical data during bridging were collected for comparison.

**Results:**

66.7% of the 24 patients survived to discharge, with mortality linked to younger age (*p* = 0.034), higher pre-ECMO pSOFA scores (*p* = 0.019), and congenital heart disease. ECMO cannulation was mostly peripheral (66.7%), with left heart decompression in 87.5%. External cardiopulmonary resuscitation (50% of cases) increased mortality risk (*p* = 0.027). The death group had higher peak/trough/average pSOFA scores, reinforcing its predictive value. Non-survivors had more complications (ECMO reuse, septic shock, neurological issues) after heart transplant. pSOFA trends distinguished outcomes: survivors showed declining scores (*p* = 0.006), and average pSOFA ≤8 predicted better survival (*p* = 0.003). ECPR patients had worse baselines but might recover with optimized management. Findings support pSOFA-guided risk stratification in ECMO-bridged HTx.

**Conclusion:**

Continuous pSOFA monitoring effectively risk-stratifies ECMO-bridged pediatric transplant candidates, identifying high-risk patients after transplant. Planned ECMO initiation yields better outcomes than ECPR. These findings warrant prospective validation to optimize bridging strategies.

## 1 Introduction

Advanced heart failure (ADHF) is one of the leading causes of death in children with cardiovascular diseases. For children who do not respond to guideline-directed medical therapy, heart transplantation (HTx) remains the primary treatment. However, according to the Pediatric Heart Transplant Society (PHTS), the severe shortage of donor hearts leads to approximately 17% of children dying while waiting, with about 20.2–26% experiencing progressive hemodynamic deterioration requiring temporary mechanical circulatory support (tMCS) as a bridge to heart replacement therapy, including extracorporeal membrane oxygenation (ECMO)(4.7–5.7%) and ventricular assist device (VAD) (10–20.2%) (1, 2). Due to limitations in VAD size and domestic development in China, most children (especially those with low body weight) are not suitable for VAD. Union Hospital, Tongji Medical College, Huazhong University of Science and Technology is the largest institution for pediatric HTx nationwide, with 227 children undergoing HTx (including 2 re-transplant) since 2009, none of whom had used VAD as bridge to HTx. Therefore, although the use of VAD in children is increasing internationally, and studies have shown that the prognosis of VAD bridging is comparable to or better than ECMO (3, 4), and some even recommend using ECMO as a bridge-to-decision to transition to VAD (4), ECMO remains an important option for pediatric emergency bridging in short future, especially in China, due to its rapid establishment, biventricular support capability and fewer body weight restrictions.

However, compared to adult patients, pediatric populations, especially low-weight children, face unique clinical challenges, including the complexity and technical demands of cannulating, precise anticoagulation management, a higher proportion of neurological complications (10–20%) and infection risks due to immature immune systems (5–8). These factors make perioperative ECMO management a critical factor affecting transplant outcomes in these children. Multiple studies have shown that the transplant prognosis of children bridged with ECMO is significantly worse than that of children transplanted directly (3, 9), and children implanted under external cardiopulmonary resuscitation (ECPR) face the additional challenge of impact of cardiac arrest and low hemoperfusion. When ECMO initiation seems to be unavoidable, determining the optimal implantation timing, optimizing recipient status during bridging, and early identification of risk factors may improve outcomes for these children.

The pediatric sequential organ failure assessment (pSOFA) score was proposed in 2017 by Matics et al. (10) as an adaptation of the adult SOFA score, adjusted for age to create a pediatric version, covering respiratory, circulating, hepatic, renal, coagulating and neurological systems. Originally used to evaluate organ status in septic shock, its comprehensive system coverage and continuous scoring have led many to apply it to assess organ function and prognosis in other critically ill children (11–14), and it has gained increasing popularity. Currently, pSOFA has not been used to evaluate the status of children supported by ECMO, especially those bridged to HTx. This study retrospectively reviewed children who underwent HTx at our center, using pSOFA to assess their organ function before and during ECMO bridging, in order to guide ECMO management and prognosis prediction.

## 2 Materials and methods

### 2.1 Research objectives and study population

This research was designed to compare the peri-bridge and peri-heart transplantation clinical characteristics of ECMO-bridged pediatric candidates with different outcomes, and to explore whether pSOFA has the ability to differentiate between different groups. We screened 188 patients under 18 years of age who underwent HTx at Union Hospital, Tongji Medical College, Huazhong University of Science and Technology, from January 2018 to April 2025, identifying 26 children who received veno-arterial ECMO (VA-ECMO) for bridging preoperatively. Two children who underwent ECMO bridging for retransplantation due to primary graft dysfunction (PGD) were excluded, leaving 24 cases included. Cases before 2018 were not considered for analyzing because of missing data and possible heterogeneity of medical protocols, as well as no application of ECMO for bridging. Children discharged in a moribund state with parents refusing further medical care were considered dead in hospital. The children were divided into two groups based on their living status while discharged, and the clinical characteristics of the two groups during the perioperative period, especially during ECMO support, were compared, which included pre-ECMO echocardiography results, blood test data, ECMO cannulating protocol and complications, as well as transplant surgery duration, donor characteristics, postoperative support, complications, hospital stay, and costs. pSOFA scores were collected once before ECMO and daily after ECMO implantation till the day before transplant. Few children underwent right heart catheterization for pulmonary artery pressure (PAP) measurement, which therefore was not included in this research. The study was approved by the Ethics Committee of Union Hospital, and all data were collected from the hospital’s medical record system.

### 2.2 ECMO implantation indications

The decision to implant ECMO was made jointly by cardiovascular surgeons, cardiac ICU physicians, and perfusionists. Inability to maintain circulation under standard cardiopulmonary resuscitation was an absolute indication for ECMO implantation, known as ECPR. However, if the patient’s circulation could barely be maintained with vasoactive drugs, whether to implant ECMO was controversial. Most centers relied on the comprehensive judgment of multiple physicians based on experiences to determine the need for ECMO. At our center, the primary indications for planned ECMO implantation included progressive oliguria, liver injury, severe arrhythmias, and gradually increasing doses of inotropic drugs, but there was no absolute cutoff value.

### 2.3 ECMO implantation and transplant protocol

Among the 24 children, 4 were transferred from other hospitals on ECMO. We obtained their ECMO implantation timing and methods, as well as whether they were implanted under ECPR, from medical records, but pre-implantation test results were unavailable. The remaining children underwent ECMO implantation at our center, with some receiving left heart decompression to alleviate left ventricular preload and pulmonary edema. For centrally cannulated children, venous cannulas were placed in the right atrium, with or without left atrial cannulation, and arterial cannulas were placed in the aorta. For peripherally cannulated children, most were cannulated via the right carotid artery and jugular vein, with 2 children cannulated via the right femoral vessels, with or without atrial septal shunt devices (aperture 4–10 mm). One child underwent a hybrid cannulation method with aortic cannulation and femoral vein/superior vena cava cannulation. All children were intubated before ECMO implantation and failed to wean from ECMO before transplantation. The surgery were all performed using the bicaval orthotopic heart transplantation method. Donor hearts were evaluated and collected by the cardiac surgeons in our department, and postoperative management was performed by the same team.

### 2.4 General ECMO management

All children initially received heparin anticoagulation with monitoring of coagulation function, aiming to maintain ACT at 180–200 s and APTT at 1.5–2 times the normal value. One child was switched to argatroban due to significant heparin induced thrombocytopenia (HIT), and another was switched to argatroban combined with low-molecular-weight heparin due to rapidly developing ECMO thrombosis despite high-dose heparin in the first 24 h. MAP was maintained at 50–70 mmHg according to age, with dopamine, dobutamine, epinephrine, captopril, or nitroprusside used for blood pressure control as needed. Aortic valve opening was observed with echocardiography in all children during ECMO support. Tracheal extubation was performed in children with relatively stable neurological and pulmonary conditions. Children who could not be extubated received intermittent sedation with dexmedetomidine and fentanyl, with neurological function assessed during sedation pauses.

### 2.5 Statistical methods

Measurement data are expressed as median (IQR) and analyzed using the Mann–Whitney U test while categorical variables are expressed as percentages (%)and analyzed using the chi-square test or Fisher’s exact test. Survival analysis was performed using the Breslow test and Kaplan–Meier survival curves. Statistical significance was defined as *p* < 0.05. All statistical analyses and figure plotting were performed using GraphPad Prism 10.3.1.

## 3 Results

### 3.1 Clinical characteristics during perioprative period

**Figure 1:**
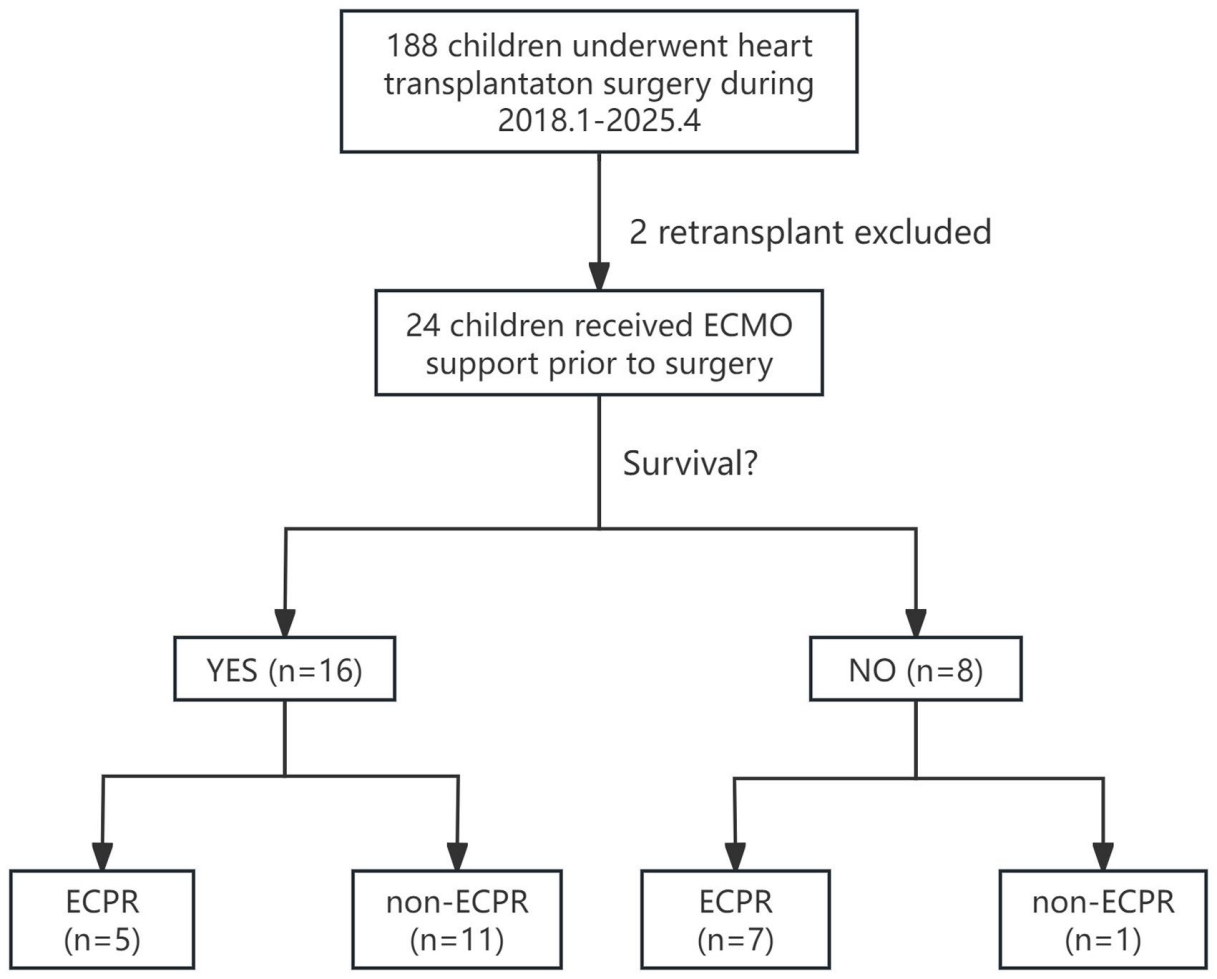
Enrollment, grouping, and in-hospital outcomes follow-up for children who received ECMO as a bridge to heart transplant. ECMO, extracorporeal membrane oxygenation; ECPR, extracorporeal membrane oxygenation.

This study included 24 children under 18 years of age who underwent VA-ECMO bridging for orthotopic heart transplantation at our center from January 2018 to April 2025, all of whom were assessed as INTERMACS level 1 or 2. Children who received retransplantation during hospitalization were excluded (Figure 1). Sixteen children (66.7%) were alive while discharged, and eight (33.3%) were not. Among them, 12 were male (50%), with the youngest aged 2 years and the oldest 14 years, the lowest weight 10 kg, and the highest weight 55 kg. The median BMI was 13.72 (13.04, 15.95) kg/cm^2^. The predominant cause of ADHF was cardiomyopathy (70.8%), including dilated cardiomyopathy (DCM) in 14 children (58.3%) and hypertrophic cardiomyopathy (HCM) in 3 (12.5%), followed by congenital heart disease (CHD) in 4 (16.7%). The longest ECMO support duration was 32 days, and the shortest was 1 day. Most underwent echocardiography and necessary blood tests before ECMO implantation, with no significant differences in baseline characteristics. However, data showed the death group had relatively younger age (*p* = 0.034) and higher pSOFA score before ECMO initiation (*p* = 0.019) compared to the survival group (Table 1) with an overall median score of 8.5 (6, 12). Besides, heart congenital heart disease appeared to be a risk factor for death after ECMO-bridged HTx.

**Table 1:** Clinical characteristics prior to surgery (before ECMO) grouped by living status.

Variables	Total (*n* = 24)	Survival		*p*-value
		Y (*n* = 16)	N (*n* = 8)	
Male, n (%)	12(50)	10(62.5%)	2(25%)	0.193
Age, (y)	7(4.25, 11.0)	10(6.25, 11)	5(2.75, 7)	0.034
BMI,(kg/cm2)	13.72(13.04, 15.95)	13.70(13.04, 16.46)	13.96(13.33, 14.90)	0.903
Diagnosis				
Dilated cardiomyopathy	14(58.3)	11(68.8)	3(37.5)	0.204
Hypertrophic cardiomyopathy	3(12.5)	3(18.8)	0(0)	0.526
Congenital heart disease	4(16.7)	0(0)	4(50)	0.007
Valvular heart disease	1(4.2)	1(6.25)	0(0)	1.000
Other	2(8.3)	1(6.25)	1(12.5)	1.000
Cardiac surgery history, n (%)	5(20.8)	2(12.5)	3(37.5)	0.289
Before ECMO				
WBC (x10^12/L)	10.18(7.69, 13.69)	7.93(7.3, 11.94)	13.00(11.31, 19.06)	0.269
Hb (g/L)	116(103, 126)	109.5(99.25, 123.5)	118(105.5, 131)	0.315
Hct (%)	34.8(32.7, 39.7)	36.3(32.95, 40.3)	34.1(31.9, 37.5)	0.339
PLT (x10^9/L)	228(144, 270)	228(178, 321.5)	216.5(123.25, 252)	0.315
TBil (μmol/L)	34.9(21.2, 61.55)	34.9(25.3, 55.4)	33.8(14.98, 60.43)	1.000
DBil (μmol/L)	13.7(8.45, 30.9)	13.4(8.1, 23.55)	25(10.4, 44)	0.533
ALT (U/L)	71(34, 270.5)	113(47.280)	50(20, 83)	0.219
AST (U/L)	63(39.5, 412.5)	68(52, 584)	46(38, 51)	0.136
Alb (g/L)	37.5(32.8, 40.25)	37.5(32.5, 40.4)	37.45(34.95, 39.35)	0.895
Cr (μmol/L)	55.9(38.05, 77.65)	58(43.6, 87.6)	39.35(35.08, 59.9)	0.254
BUN (mmol/L)	7.3(5.85, 12.79)	6.78(5.86, 13.66)	7.72(6.01, 9.91)	0.861
INR	1.48(1.295, 2.58)	2.49 (1.32, 2.83)	1.39(1.29, 1.53)	0.195
APTT (s)	40.3(36.95, 45.35)	44.4 (36.9, 48.8)	39.95(37.65, 41.95)	0.346
NTproBNP (pg/mL)	16,150(11,075, 22,150)	15,600(11,075, 21,900)	18,100(12447.5, 23,375)	0.777
Lac (mmol/L)	8.6 (5.65, 12.18)	8.4 (6.3, 10.3)	11.7(7.45, 12.65)	0.840
LVEF (%)	27.6 (18.0, 38.0)	24.6 (17.75, 29.25)	34(20, 45)	0.118
pSOFA	8.5 (6, 12)	6.5 (6, 8.75)	12(11.25, 15)	0.019

Continuous data are presented as the median (IQR). Categorical data are presented as counts (%). ECMO, extracorporeal membrane oxygenation; BMI, body mass index; DCM, dilated cardimyopathy; HCM, hypertrophic cardimyopathy; CHD, congenital heart disease; Valvular heart disease; WBC, white blood cell; Hb, hemoglobin; Hct, hematocrit; PLT, platelet; TBIL, total bilirubin; DBIL, direct bilirubin; ALT, alanine aminotransferase; AST, aspartate aminotransferase; Alb, albumin; Cr, serum creatinine; BUN, blood urea nitrogen; INR, international normalized radio; APTT, activated partial thromboplastin time; NTproBNP, N-terminal pro-brain natriuretic peptide; Lac, lactate; LVEF, left ventricular ejection fraction; pSOFA, pediatric Sequential Organ Failure Assessment.

Characteristics of the 24 children during ECMO bridging were showed in Table 2, none of whom were successfully weaned from ECMO before transplantation. Right jugular vessels for ECMO cannulation were firstly used at our center in 2021. Before that, central cannulating was widely used for most children, while femoral vessels were typically used for teenagers and adults. Among the 24 children, 16 (66.7%) underwent peripheral cannulation, and 21 (87.5%) received left heart decompression, including left atrial cannulation in 8 (33.3%) and atrial septostomy in 13 (54.2%). Half of them were implanted under ECPR with data indicating ECPR as a risk factor for in-hospital death (*p* = 0.027). Comparing the incidence of pulmonary edema and lung infection between the two decompression methods, atrial septostomy was slightly inferior to direct cannulating in left heart (*p* = 0.056), with 3 children who did not undergo left heart decompression were excluded from comparison due to the small sample size. There were no significant differences between the two groups in the worst values of blood test results during bridging and ECMO support duration [median ECMO duration: 8 (4.75, 14)days]. The incidence of arrhythmias, pulmonary complications, renal complications (requiring dialysis), hemorrhage/thrombosis and neurological complications (including delayed awakening, seizures, delirium, and amorphous type)during bridging also showed no notable differences. However, the death group had a significantly higher peak, trough and average pSOFA value, which showed greater potential for outcomes prediction than single indicators.

**Table 2:** Clinical characteristics during ECMO bridging grouped by living status.

Variables	Total (*n* = 24)	Survival		*p*-value
		Y (*n* = 16)	N (*n* = 8)	
ECPR, n (%)	12(50)	5(31.25)	7(87.5)	0.027
Peripheral cannulation, n (%)	16(66.7)	11(68.8)	5(62.5)	1.000
LA decompression, n (%)	21(87.5)	15(93.8)	6(75.0)	0.249
During ECMO				
Trough Hb (g/L)	78(66.75, 84.25)	80.5(72.25, 86.75)	70.5(61.25, 78.75)	0.125
Trough PLT (x10^9/L)	52.5(35.0, 98.75)	53(43, 97)	40(20.75, 112.5)	0.540
Peak TBil (μmol/L)	66.4(31.83, 133.8)	66.4(33.45, 118.13)	92(31.83, 175.7)	0.540
Peak DBil (μmol/L)	34(17.03, 71.58)	30.25(16.45, 45.6)	82.8(33.1, 122.08)	0.090
Peak ALT (U/L)	150(60.75, 1135.5)	244.5(61.5, 1135.5)	113(52.25, 722.25)	0.582
Peak AST (U/L)	379.5(183.75, 2282.75)	379.5(193.25, 2282.75)	545(54.5, 2230.75)	0.806
Trough Alb (g/L)	32.45(31.58, 36.45)	32.4(31.32, 95)	36.7(32.38, 38.88)	0.057
Peak Cr (μmol/L)	66.25(52.33, 101.45)	68.15(53.5, 128.5)	64.75(45.58, 86)	0.298
Peak BUN (mmol/L)	9.87(7.97, 14.04)	11.65(8.85, 14.04)	9.04(7.12, 11.08)	0.327
Peak NTproBNP (pg/mL)	19,700(12,000, 30,000)	17,700(12,100, 30,625)	30,000(19,915, 30,000)	0.885
Peak Lac (mmol/L)	6.3(3.3, 15.15)	5.65(2.95, 11.55)	16(3.8, 20)	0.284
Peak pSOFA	9.5(8, 12)	8.5(7, 10)	12(11.75, 12)	0.004
Trough pSOFA	7(3.75, 8.25)	7(3.75, 13)	8(7, 9.75)	0.008
Average pSOFA	8.73(6.72, 10)	7.18(5.48, 8.81)	10.23(9.7, 11.4)	0.003
ECMO time (d)	8(4.75, 14)	4.75(8, 11)	4.75(10, 14.25)	0.712
Complications				
Arrhythmia	12(50.0)	8(50.0)	4(50.0)	1.000
Respiratory system	16(66.7)	11(68.8)	5(62.5)	1.000
Liver damage	18(75.0)	12(75.0)	6(75.0)	1.000
Hemo−/peritoneal dialysis	9(37.5)	4(25.0)	5(62.5)	0.099
Hemorrhage or thrombus	14(58.3)	10(62.5)	4(50.0)	0.673
Abnormal mental status	8(33.3)	3(18.8)	5(62.5)	0.065

ECMO, extracorporeal membrane oxygenation; LA, left atrium; HTx, heart transplantation; Hb, hemoglobin; Hct, hematocrit; PLT, platelet; TBIL, total bilirubin; DBIL, direct bilirubin; ALT, alanine aminotransferase; AST, aspartate aminotransferase; Alb, albumin; Cr, serum creatinine; BUN, blood urea nitrogen; BNP, N-terminal pro-brain natriuretic peptide; Lac, lactate; pSOFA, pediatric Sequential Organ Failure Assessment.

Table 3 showed datas of peri-HTx period. All children were mechanically ventilated before transplantation with a median mechanical ventilation time of 120 (58,215) hours, and 21 children (87.5%) were extubated before the surgery, with no significant difference between the two groups. The median donor age was 16 (12.26, 27.5) years, with 13 (54.2%) being male. The median donor-recipient weight ratio (DRWR) was 1.97 (1.45, 2.42), and the median donor-recipient height ratio (DRHR) was 1.23 (1.08, 1.38), which slightly exceeded the guideline-recommended range of 0.7–1.2 (15) due to donor scarcity. The median cold ischemia time (CIT) for donor hearts was 323 (288.25, 346) minutes, with the median surgical time 300 (262.5, 325) minutes, the median cardiopulmonary bypass (CPB) time 118 (103,131) minutes and the median aortic cross-clamp time 29 (25, 34) minutes. ECMO was weaned intraoperatively in 22 children (91.7%), with no statistically differences between the two groups. But the death group fared worse in many aspects after HTx, including higher delayed chest closure (*p* = 0.021) rate, reintubation rate (*p* = 0.028), ECMO reuse rate (*p* < 0.001), dialysis rate (*p* = 0.001), septic shock rate (*p* = 0.028) and neurological complication rate (*p* = 0.027), as well as longer mechanical ventilation time (*p* = 0.002) and post operative costs (*p* = 0.028).

**Table 3:** Perioperative clinical characteristics and short-term outcomes grouped by living status.

Variables	Total (*n* = 24)	Survival		*p*-value
		Y (*n* = 16)	N (*n* = 8)	
Pre-op MV duration (h)	120(57, 215)	93.75(44, 163.5)	179(123, 356.25)	0.082
Pre-op extubation, n (%)	13(54.2)	10(62.5)	3(37.5)	0.390
Intraoperative conditions				
Donor age (y)	16(12.26, 27.5)	16(14, 28)	15(5.75, 23)	0.357
Male donor, n (%)	13(54.2)	11(68.8)	2(25.0)	0.082
DRWR	1.97(1.45, 2.42)	1.94(1.54, 2.10)	2.33(1.45, 3.21)	0.504
DRHR	1.23(1.08, 1.38)	1.22(1.08, 1.33)	1.42(1.36, 1.42)	0.483
Donor heart CIT(min)	323(288.25, 346)	326(297.5, 336)	301(257.75, 409.75)	0.602
Operation time(min)	300(262.5, 325)	285(265, 300)	312(255, 373.5)	0.690
CBP time(min)	118(103, 131)	107(103, 122)	126.5(118, 153.25)	0.118
Aorta clamp time(min)	29(25, 34)	29(24, 32)	30(25, 36.25)	0.69
Weaning from ECMO, n(%)	22(91.7)	16(100.0)	6(75.0)	0.101
Post-operative conditions				
Delayed chest closure, n(%)	7(58.3)	2(12.5)	5(62.5)	0.021
Post-op MV duration(h)	93.75(48.375, 531.125)	52(38.5, 108.75)	610.5(429.13, 862.13)	0.002
Re-intubation, n(%)	5(20.8)	1(6.3)	4(50.0)	0.028
Pulmonary infection, n(%)	14(58.3)	8(50)	6(75)	0.388
Positive sputum culture, n(%)	21(87.5)	14(87.5)	7(87.5)	1.000
Post-op ECMO, n(%)	6(25)	0(0)	6(75.0)	0.000
Hemo−/peritoneal dialysis, n(%)	9(37.5)	2(12.5)	7(87.5)	0.001
Abnormal mental status, n(%)	12(50)	5(31.3)	7(87.5)	0.027
Septic shock, n(%)	3(12.5)	0(0)	3(37.5)	0.028
ICU stay(d)	20.5(13.75, 27)	18.5(13.75, 25.25)	26(17.25, 52.75)	0.327
Hospital stay(d)	47(31.5, 57.5)	49(40.5, 57.5)	26(17.25, 52.75)	0.071
Post-op costs(k¥)	383.46(261.00, 846.05)	330.71(239.25, 407.27)	625.62(578.4, 1204.82)	0.028
1-month survival	19(79.2)	14(87.5)	5(62.5)	0.289

Op, operation; MV, mechanical ventilation; DRWR, donor-receptor weight ratio; DRHR, donor-receptor height ratio; CIT, cold ischemia time; CPB, cardiopulmonary bypass; ECMO, extracorporeal membrane oxygenation; ICU, intensive care unit.

### 3.2 Application of pSOFA score for outcome predicting

**Figure 2:**
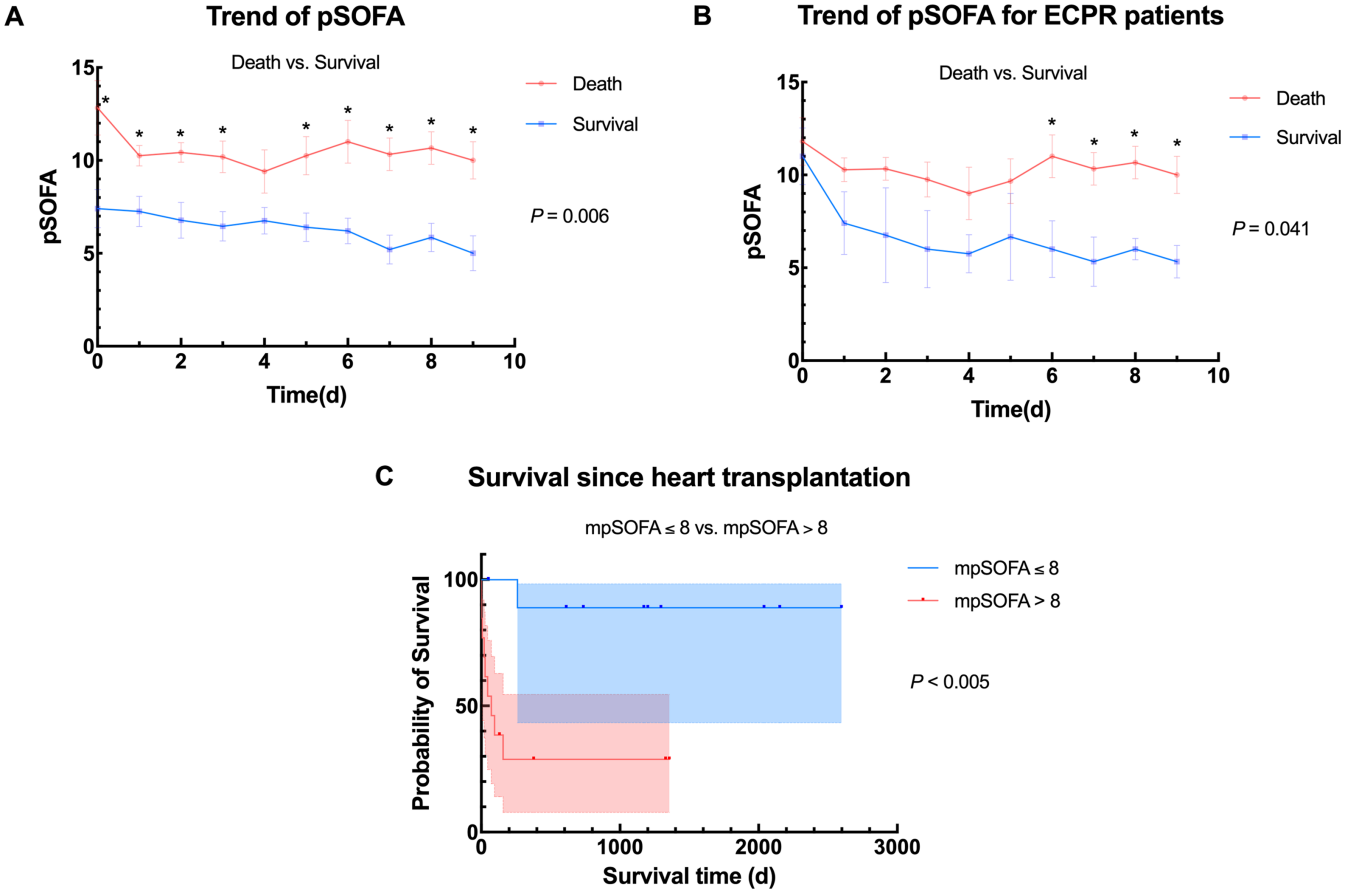
Trends of pSOFA and survival between different populations. **(A)** Overall trend of pSOFA between death group and survival group. Average daily pSOFA were significantly different (*p* = 0.006); **(B)** Trend of pSOFA between death group and survival group in ECPR patients. Average daily pSOFA were significantly different (*p* = 0.041); **(C)** Comparison of survival between different stratification according to average pSOFA during bridging (mpSOFA ≤ 8 vs. mpSOFA > 8, *n* = 11 vs. 13), pSOFA, pediatric Sequential Organ Failure Assessment; mpSOFA, average pediatric Sequential Organ Failure Assessment; ECPR, extracorporeal membrane oxygenation; ECMO, extracorporeal membrane oxygenation.

Daily pSOFA scores were recorded during bridging until the day before transplantation, 20 children (83.3%) receiving donor hearts within 2 weeks. pSOFA scores of the 2 groups from day 0 (before ECMO) to day 9 (datas were insufficient for calculating after day 9) were plotted against bridging time (Figure 2A), Addressed as pSOFA0-9, revealing a general downward trend in survival group, with significant differences of daily average pSOFA compared to the death group (*p* = 0.006) throughout bridging duration. For ECPR patients, the trend also showed statistically difference after day 6 (Figure 2B), which suggests that patients who have better prognosis after HTx may already have signs during a short term of ECMO bridging no matter whether they were bridged under ECPR. With 8.73 being the median value of pSOFA score through out bridging, we choose 8 as a cutoff to divide the 24 children into two groups based on their average pSOFA scores (mpSOFA) throughout bridging duration (mpSOFA≤8 vs. mpSOFA>8). Kaplan–Meier curves showed significantly better postoperative survival in children with lower mpSOFA (*p* = 0.003), indicating that pSOFA scores during ECMO bridging have great potential in predicting transplant prognosis (Figure 2C).

### 3.3 pSOFA in comparison of whether ECPR or not

**Figure 3:**
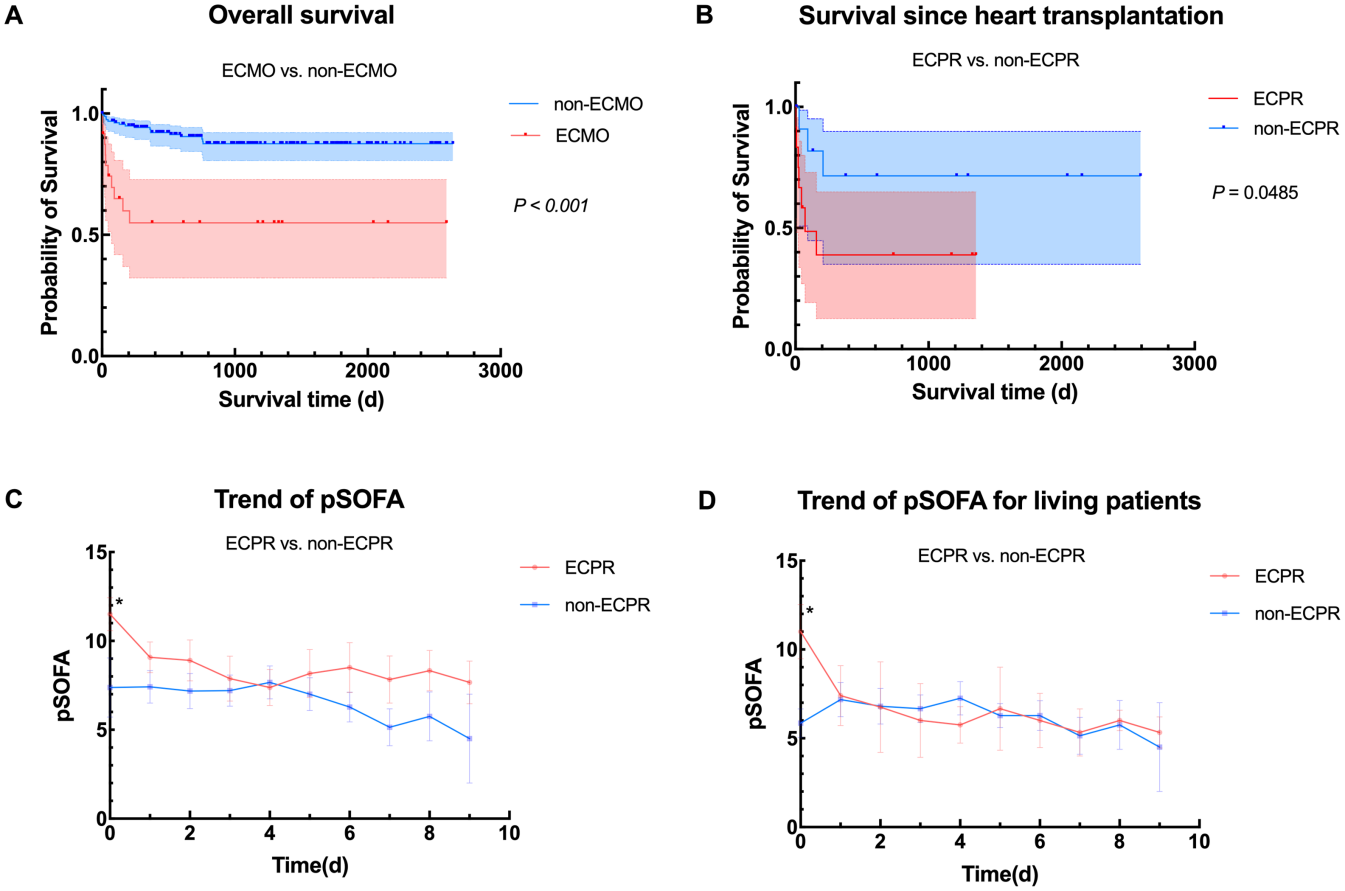
Comparison between ECPR and non-ECPR. **(A)** Overall survival in heart transplant children with or without ECMO support from January 2018 to April 2025 (*n* = 24 vs. 162); **(B)** Comparison of survival between different ECMO implanting status (ECPR vs. non-ECPR, *n* = 12 vs. 12); **(C)** Overall trend of pSOFA between ECPR group and non-ECPR group. pSOFA before ECMO initiation (pSOFA0) were significantly different (*p* = 0.020); **(D)** Trend of pSOFA between ECPR group and non-ECPR group in living patients. pSOFA before ECMO initiation were significantly different (*p* = 0.028). ECMO, veno-arterial extracorporeal membrane oxygenation; pSOFA, pediatric Sequential Organ Failure Assessment; ECPR, extracorporeal cardiopulmonary resuscitation.

While it is well known that ECMO-bridged children have worse prognosis than non-ECMO children, consistent with our center’s experience (*p* < 0.001) (Figure 3A), patients undergoing ECPR also shows poorer outcomes according to Figure 3B, as was expected. Supplementary Tables 1–3 demonstrates the differences when grouped by ECPR or not, also showing poorer pre-ECMO indicators and worse outcomes. Interestingly, children with planned ECMO implantation had lower preoperative left ventricular ejection fraction (LVEF) (*p* = 0.011). This may be because the non-ECPR group included more children with DCM, while other types of ADHF may not primarily present with reduced LVEF. Alternatively, lower LVEF may prompt doctors to be more vigilant and initiate mechanical support more aggressively.

To further investigate if pSOFA score may also work in telling patients under ECPR from those were not, we plotted Figures 3C,D, which turned out that only pSOFA0 showed notable differences in overall and the living population, implying that although being a risk factor, the reversible adverse effects of ECPR may gradually alleviated during ECMO support due to improved circulation and optimized management, even approaching non-ECPR patients. To sum up, ECPR patients are inferior to non-ECPR patients in terms of their status before ECMO bridging, late recovery and outcomes. However, if management strategy is well modified during ECMO bridging and key organ function is maintained under the guidance of serial scoring, the damage caused by heart arrest and low-flow status may be made up.

## 4 Discussion

This study summarizes the perioperative characteristics of pediatric heart transplant candidates bridged with ECMO for heart transplantation and introduces pSOFA scoring during ECMO support, which suggests that mpSOFA>8 and ECPR may predict poorer outcomes, highlighting the potential of pSOFA application and significance of organ protection during ECMO bridging, maybe also the advantages of planned ECMO implantation. We are the first to validate pSOFA’s predictive potential in pediatric ECMO-bridged heart transplantation, extending its application to mechanical support and organ transplantation.

Besides pSOFA, the Pediatric Risk of Mortality (PRISM) (16) and Pediatric Logistic Organ Dysfunction-2 (PELOD-2) (17) scores are also widely used pediatric scoring systems. PRISM predicts mortality based on the worst indicators within 24 h of PICU admission, while PELOD-2 assesses organ failure at specific time points (day 1, 2, 5, 8, 12, 16, 18, and PICU discharge) across five systems (neurological, cardiovascular, respiratory, platelet, and renal) (17). In contrast, pSOFA focuses on daily continuous monitoring of organ function changes. ADHF in children leads to systemic hypoperfusion, affecting multiple organs and vice versa. Organs may maintain delicate balance for extended periods or deteriorate rapidly, but donor heart availability is unpredictable. For such children with prolonged but unstable conditions, PRISM’s one-time scoring may be insufficient, and even modified versions PRISM-III (18) also lack comprehensive and continuous monitoring.

What’s more, in cardiac dysfunction, especially right heart failure, liver injury (e.g., hepatomegaly, elevated bilirubin) may emerge early and correlate closely with prognosis. Bilirubin levels twice the normal limit are even considered a relative contraindication for HTx (15, 19–21). pSOFA incorporates bilirubin monitoring, and its daily scoring enables physicians to detect trends more clearly, identify risk factors earlier, and prioritize organ protection, especially for liver. By categorizing children during ECMO support into high-risk (mpSOFA>8) and low-risk (mpSOFA≤8) groups, with the former having significantly worse prognoses, pSOFA scoring may guide targeted organ support, optimize transplant waiting lists, inform decisions on ECMO weaning or transition to VAD, adjust postoperative management, and help parents establish realistic expectations early.

The pSOFA score was originally designed for critically ill children with infections and did not account for mechanical support (10). In this study, ECMO improved circulatory function, potentially leading to underestimation of cardiovascular scores based on mean arterial pressure (MAP) and vasoactive drug requirements. Additionally, ECMO, which provides continuous non-pulsatile flow, may further reduce pulse pressure and potentially affect other organs (22, 23). Future studies could refine scoring weights based on MCS-specific considerations.

Due to incontinuous and incomplete pre-ECMO medical records, we only performed single-time pSOFA scoring before ECMO implantation, limiting its utility in guiding implantation timing. But it has highlighted the importance of avoiding ECPR in children awaiting transplantation. ECMO indications remain somewhat subjective and ambiguous (7), and concerns about hemolysis and anticoagulation challenges often lead to hesitation and delayed implantation. This study shows that ECPR children had higher pre-implantation lactate levels and pSOFA0 scores, suggesting that earlier ECMO initiation may be warranted when organ function shows trend of deterioration. For ADHF children, initiating continuous pSOFA monitoring upon admission could provide objective data to guide ICU transfer or MCS decisions.

As a single-center retrospective study with a small sample size, this research lacks multivariate regression statistics, pre-ECMO organ function assessments and does not fully explore systemic effects of ECMO, leading to limitations especially for patients with even more complicated post-transplant physiology. Future multi-center prospective studies incorporating mechanical support characteristics, lactate levels, nutritional status, and growth indicators could refine scoring systems, improve risk stratification, enhance high-risk patient monitoring, and optimize perioperative management in pediatric heart transplantation.

## 5 Conclusion

In summary, our study demonstrated that continuous pSOFA monitoring may effectively risk-stratifies ECMO-bridged pediatric transplant candidates, identifying high-risk patients (mpSOFA>8), and planned ECMO initiation yields better outcomes than ECPR. These findings warrant prospective validation to optimize bridging strategies.

## Data Availability

The raw data supporting the conclusions of this article will be made available by the authors, without undue reservation.
