# Supplementary material for: Pediatric sequential organ failure assessment for predicting outcomes in ECMO-bridged pediatric heart transplant recipients: experience from the largest pediatric heart transplant center in China
Source: Front Med (Lausanne). 2025 Jul 4;12:1631616. doi: 10.3389/fmed.2025.1631616 (PMC12271090; doi:10.3389/fmed.2025.1631616)
Supplement: Supplementary file 1 [file Table_1.docx]

Supplementary Material

**Supplementary Table 1|** Clinical characteristics prior to surgery grouped by implantation status.

| **Variables** | **Total（n=24）** | **ECPR** | | ***p*-value** |
| --- | --- | --- | --- | --- |
|  |  | **Y(n=12)** | **N(n=12)** |  |
| Male,n(%) | 12(50) | 6(50) | 6(50) | 1.000 |
| Age(y) | 7(4.25,11.0) | 7(3.75,10.25) | 7(4.25,11.0) | 0.468 |
| BMI(kg/cm^2^) | 13.72(13.04,15.95) | 13.63(13.04,14.73) | 13.72(13.04,15.95) | 0.488 |
| **Diagnosis** |  |  |  |  |
| DCM | 14(58.3) | 4(33.3) | 10(83.3) | 0.036 |
| HCM | 3(12.5) | 3(25) | 0(0) | 0.217 |
| CHM | 4(16.7) | 1(8.3) | 0(0) | 1.000 |
| VHD | 1(4.2) | 3(25) | 1(8.3) | 0.590 |
| Other | 2(8.3) | 1(8.3) | 1(8.3) | 1.000 |
| Cardiac surgery history,n(%) | 5(20.8) | 3(25) | 2(16.7) | 1.000 |
| **Before ECMO** |  |  |  |  |
| WBC(x10^12^/L) | 10.18(7.69,13.69) | 10.84(7.78,13.28) | 8.37(7.47,16.09) | 0.923 |
| Hb(g/L) | 116(103,126) | 123(116,133) | 105.5(101,116) | 0.123 |
| Hct(%) | 34.8(32.7,39.7) | 38.4(34.8,39.7) | 32.95(31.7,35.58) | 0.092 |
| PLT(x10^9^/L) | 228(144,270) | 254(196,270) | 219(137.5,242.75) | 0.441 |
| TBil(μmol/L) | 34.9(21.2,61.55) | 28.45(15.7,33.8) | 55.4(34.9,78.6) | 0.055 |
| DBil(μmol/L) | 13.7(8.45,30.9) | 13.4(10.4,19) | 28.1(8.1,48.9) | 0.368 |
| ALT(U/L) | 71(34,270.5) | 64.5(28.25,279.25) | 179(58,1230) | 0.086 |
| AST(U/L) | 63(39.5,412.5) | 48.5(26.75,83) | 197(52,2611) | 0.141 |
| Alb(g/L) | 37.5(32,8,40.25) | 38.8(36.38,40.33) | 34.2(32,37.6) | 0.178 |
| Cr(μmol/L) | 55.9(38.05,77.65) | 48.6(35.08,64) | 67.7(43.6,114.8) | 0.165 |
| BUN(mmol/L) | 7.3(5.85,12.79) | 7.04(5.88,9.31) | 11.91(5.86,22.7) | 0.514 |
| INR | 1.48(1.30,2.58) | 1.34(1.27,1.68) | 2.49(1.45,2.75) | 0.203 |
| APTT(s) | 40.3(36.95,45.35) | 38.3(35.95,41.23) | 44.4(40,48.2) | 0.083 |
| NTproBNP(pg/mL) | 16150(11075,22150) | 14850(9372.5,20050) | 19750(12475,28150) | 0.366 |
| Lac(mmol/L) | 8.6(5.65,12.18) | 11.7(9.35,14.8) | 6.5(3.4,8.8) | 0.030 |
| LVEF(%) | 27.6(18.0,38.0) | 34(25.4,45) | 18.5(16.93,28.25) | 0.011 |
| pSOFA | 8.5(6,12) | 12(10.25,12.25) | 6(5.75,7.5) | 0.020 |

*Continuous data are presented as the median (IQR). Categorical data are presented as counts (%).ECMO, extracorporeal membrane oxygenation; BMI, body mass index; DCM, dilated cardimyopathy; HCM, hypertrophic cardimyopathy; CHD, congenital heart disease; Valvular heart disease; WBC, white blood cell;Hb, hemoglobin; Hct, hematocrit; PLT, platelet; TBIL, total bilirubin;DBIL, direct bilirubin; ALT, alanine aminotransferase; AST, aspartate aminotransferase; Alb, albumin; Cr, serum creatinine; BUN, blood urea nitrogen; INR, international normalized radio; APTT, activated partial thromboplastin time; NTproBNP, N-terminal pro-brain natriuretic peptide; Lac, lactate;LVEF, left ventricular ejection fraction; pSOFA, pediatric Sequential Organ Failure Assessment.*

**Supplementary Table 2 |** Clinical characteristics during ECMO bridging grouped by implantation status.

| **Variables** | **Total（n=24）** | **ECPR** | | ***p*-value** |
| --- | --- | --- | --- | --- |
|  |  | **Y(n=12)** | **N(n=12)** |  |
| periphreral cannulation,n(%) | 16(66.7) | 7(58.3) | 9(75) | 0.123 |
| LA decompression,n(%) | 21(87.5) | 10(83.3) | 11(91.7) | 0.226 |
| **During ECMO** |  |  |  |  |
| trough Hb(g/L) | 78(66.75,84.25) | 78(62.75,79.5) | 82(72.25,85.75) | 0.272 |
| trough PLT(x10^9^/L) | 52.5(35.0,98.75) | 44(28.5,105.5) | 62.5(45.75,97) | 0.644 |
| peak TBil (μmol/L) | 66.4(31.825,133.8) | 50.2(27.75,94.7) | 97(40.25,133.8) | 0.453 |
| peak DBil (μmol/L) | 34(17.03,71.58) | 26.4(15.38,72.25) | 40.8(22.25,65.73) | 0.792 |
| peak ALT (U/L) | 150(60.75,1135.5) | 108(63.75,217) | 526(53.5,1899.75) | 0.525 |
| peak AST (U/L) | 379.5(183.75,2282.75) | 374.5(183.75,857.75) | 433.5(175,2375.5) | 0.817 |
| trough Alb (g/L) | 32.45(31.58,36.45) | 32.4(31.575,36.45) | 32.7(31.75,36.28) | 0.686 |
| peak Cr (μmol/L) | 66.25(52.33,101.45) | 71.4(54.95,101.3) | 61.95(52.275,107.83) | 0.795 |
| peak BUN (mmol/L) | 9.87(7.97,14.04) | 9.455(7.90,14.94) | 10.37(8.66,12.96) | 0.908 |
| peak NTproBNP (pg/mL) | 19700(12000,30000) | 30000(12900,32150) | 15950(12100,25575) | 0.451 |
| peak Lac (mmol/L) | 6.3(3.3,15.15) | 14.3(3.8,19) | 5.45(2.8,8.525) | 0.156 |
| peak pSOFA | 9.5(8,12) | 11.5(9,12) | 8.5(7.75,10.25) | 0.129 |
| trough pSOFA | 7(3.75,8.25) | 7(5.75,9.25) | 5.5(3.75,7.25) | 0.257 |
| ECMO time(d) | 8(4.75,14) | 9(5,14) | 8(4.75,14) | 0.469 |
| **Complications** |  |  |  |  |
| Arrhythmia | 12(50) | 5(41.7) | 7(58.3) | 0.660 |
| Respiratory system | 16(66.7) | 8(66.7) | 8(66.7) | 1.000 |
| Liver damage | 18(75) | 9(75) | 11(91.7) | 0.590 |
| Hemo-/peritoneal dialysis | 9(37.5) | 5(41.7) | 4(33.3) | 0.432 |
| Hemorrhage or thrombus | 14(58.3) | 7(58.3) | 7(58.3) | 1.000 |
| Abnormal mental status | 8(33.3) | 6(50) | 2(16.7) | 0.010 |

*ECMO, extracorporeal membrane oxygenation; LA, left atrium; HTx, heart transplantation; Hb, hemoglobin; Hct, hematocrit; PLT, platelet; TBIL, total bilirubin;DBIL, direct bilirubin; ALT, alanine aminotransferase; AST, aspartate aminotransferase; Alb, albumin; Cr, serum creatinine; BUN, blood urea nitrogen; BNP, N-terminal pro-brain natriuretic peptide; Lac, lactate; pSOFA, pediatric Sequential Organ Failure Assessment.*

**Supplementary Table 3 |** Perioperative clinical characteristics and short-term outcomes grouped by implantation status.

| **Variables** | **Total（n=24）** | **ECPR** | | ***p*-value** |
| --- | --- | --- | --- | --- |
|  |  | **Y(n=12)** | **N(n=12)** |  |
| Pre-op MV duration(h) | 16(66.7) | 7(58.3) | 9(75) | 0.123 |
| Pre-op extubation,n(%) | 21(87.5) | 10(83.3) | 11(91.7) | 0.226 |
| **Intra-op conditions** |  |  |  |  |
| Donor age(y) | 16(12.26,27.5) | 16(13.75,27.5) | 15.5(10,24.25) | 0.817 |
| Male donor,n(%) | 13(54.2) | 5(41.7) | 8(66.7) | 0.414 |
| DRWR | 1.97(1.45,2.42) | 2.17(1.74,2.68) | 1.73(1.41,1.98) | 0.116 |
| DRHR | 1.23(1.08,1.381) | 1.32(1.23,1.42) | 1.10(1.03,1.31) | 0.481 |
| Donor heart CIT(min) | 323(288.25,346) | 317.5(285.75,371.25) | 326.5(288.25,336) | 1.000 |
| Operation time(min) | 300(262.5,325) | 312(258.75,351) | 285(267.5,300) | 0.623 |
| CBP time(min) | 118(103,131) | 128(119.5,141.5) | 103.5(101.5,113) | 0.010 |
| Aorta clamp time(min) | 29(25,34) | 30(29,36.5) | 26(21.75,31) | 0.285 |
| Weaning from ECMO,n(%) | 22(91.7) | 11(91.7) | 11(91.7) | 1.000 |
| **Post-op conditions** |  |  |  |  |
| Delayed chest closure,n(%) | 7(58.3) | 6(50) | 1(8.3) | 0.069 |
| Post-op MV duration(h) | 93.75(48.38,531.13) | 313.25(83.63,642.38) | 52(34.75,105) | 0.021 |
| Re-intubation,n(%) | 5(20.8) | 5(41.7) | 0(0) | 0.037 |
| Pulmonary infection,n(%) | 14(58.3) | 8(66.7) | 6(50) | 0.680 |
| Positive sputum culture,n(%) | 21(87.5) | 11(91.7) | 10(83.3) | 1.000 |
| Post-op ECMO | 6(25) | 5(41.7) | 1(8.3) | 0.155 |
| Dialysis,n(%) | 9(37.5) | 7(58.3) | 2(16.7) | 0.089 |
| Abnormal mental status,n(%) | 12(50) | 8(66.7) | 4(33.3) | 0.220 |
| Septic shock,n(%) | 3(12.5) | 3(25) | 0(0) | 0.217 |
| ICU stay(d) | 20.5(13.75,27) | 23(13.75,51.25) | 19.5(13.75,25.25) | 0.544 |
| Hospital stay(d) | 47(31.5,57.5) | 47(24,58.75) | 47(32.5,57) | 0.758 |
| Post-op costs(￥) | 383458.9  (260996.9,846049.7) | 574120.9  (275280.2,960369.6) | 375426.4  (271220.3,625648) | 0.680 |
| In-hospital survival | 16(66.7) | 5(41.7) | 11(91.7) | 0.001 |
| 1 month survival | 19(79.2) | 8(66.7) | 11(91.7) | 0.004 |

*Op, operation; MV, mechanical ventilation; DRWR, donor-receptor weight ratio; DRHR, donor-receptor height ratio; CIT, cold ischemia time; CPB, cardiopulmonary bypass; ECMO, extracorporeal membrane oxygenation; ICU, intensive care unit.*
